# Report of the Diseases of Edinburgh for October, 1808

**Published:** 1808-12

**Authors:** John Roberton

**Affiliations:** Surgeon


### *Report of the Diseases of Edinburgh for October*, 1808. By John Roberton, Surgeon.

The weather for the first week of the present month was clear, and the mornings in general frosty. It then became variable, and about the middle of the month we had, during one whole day, a fall of snow. About the beginning of the last third of the month, we had some very heavy gales of wind, and till the end, the changes in the weather were were very frequent. —— The thermometer varied from 32° to 60°, but it was for the most part below 50°. The barometer, in the beginning of the month, was high, but, during the various changes of the weather, it varied considerably till about the end, when, independently of the changes, it rose to a considerable height.

During the present month, when families generally return from the country to their town residence, the healthy appearance that almost every one displays, is the clearest proof of the salubrious effects of the country air compared to that of Edinburgh, even although it is probably more healthily situated than any other city of equal extent.

On a review of the previous Reports, it will appear, that there has been no disease of any great importance peculiar to this city during the summer months; that most of the complaints have rather been in consequence of the previous winter and spring diseases, which being chiefly of an inflammatory nature, occasion diseases of local or general debility.

The bowel complaints formerly mentioned have greatly abated, nor does either scarlatina or intermittent fever seem general. Fever of the typhoid type does not seem to have abated, but on the contrary to have augmented both in frequency and severity.

We certainly are much indebted to the late Dr. Currie of Liverpool, for his valuable and accurate observations respecting the efficacy of cold in the removal of fever. It is strange, however, that, at this late period, after the nature of that practice ought so completely to be understood, we should find, as I have lately had occasion to see, the indiscriminate use of cold in every stage of the disease. On the present occasion, I had an opportunity to observe this in consequence of being called to alleviate the very unfavourable symptoms which followed its application in the cold stage. Dr. Currie's rules upon this subject are so extremely plain, that it is disgraceful in any one to mistake or misapply them. By the bye, I may mention that I have for several years past been in the habit of applying cold in a different, less formidable, and equally successful way, from that which is recommended by Dr. Currie. Concerning either affusion, immersion, or even bathing the body by means of a sponge, in some cases of fever, a very inconvenient operation, I attempted to apply the cold by means of a large bladder filled with the coldest water I could procure, and placed immediately over the region of the stomach, while a handful of common salt was dissolving in it.

The

The invariable effects of this mode of applying cold, were indeed surprisingly beneficial. In a few minutes the pulse fell, often from 120 to 90, or even fewer beats in a minute; the skin became cool, and in almost every instance sleep was induced within a very short period, often indeed before the bladder was removed from the breast. From this sleep, the patient usually awoke greatly refreshed and relieved in every respect, though perhaps for several days no such refreshing sleep had been obtained. This is certainly a more simple mode of applying cold in fevers; and I can vouch for its being as efficacious as any other recommended by medical writers.

Since the commencement of this month, inflammatory complaints have, as usual, on the approach of winter, made their appearance in various quarters of the city. There have appeared some cases of ophthalmia, some of pneumonia, and a still greater number of catarrh. The last disease, by the bye, I may mention, has, in various instances, been attended with a considerable degree of fever.

Most of the cases of ophthalmia have disappeared by the use of a solution for bathing the eyes, composed of the extract or some other preparation of lead. The pneumonia has easily yielded to bleeding, which it was seldom necessary to repeat; and the catarrh was removed by the use of cathartic medicines.

In my last Report, I mentioned a plan for preserving the feet from dampness, which there can be no question must be attended with the most complete success. I have, in two or three instances, since that period, advised such persons as were, from the nature of their employment, exposed to it, to furnish themselves with a cere-cloth shirt to be worn above their other shirt; and in every instance in which I have recommended it, the most marked benefit has been obtained. Even during the largest continued rains, they were, with the additional employment of the formerly proposed plans, preserved entirely free from wetness.

*No. 4, St. James's Street.*

